# Exploring the functional interaction between POSH and ALIX and the relevance to HIV-1 release

**DOI:** 10.1186/1471-2091-10-12

**Published:** 2009-04-24

**Authors:** Jörg Votteler, Elena Iavnilovitch, Orit Fingrut, Vivian Shemesh, Daniel Taglicht, Omri Erez, Stefan Sörgel, Torsten Walther, Norbert Bannert, Ulrich Schubert, Yuval Reiss

**Affiliations:** 1 Institute of Virology, Friedrich-Alexander University, Erlangen, Germany; 2 Proteologics Ltd, Rehovot, Israel; 3 ViroLogik GmbH, Erlangen, Germany; 4 Robert Koch Institute, Berlin, Germany

## Abstract

**Background:**

The ALG2-interacting protein X (ALIX)/AIP1 is an adaptor protein with multiple functions in intracellular protein trafficking that plays a central role in the biogenesis of enveloped viruses. The ubiquitin E3-ligase POSH (plenty of SH3) augments HIV-1 egress by facilitating the transport of Gag to the cell membrane. Recently, it was reported, that POSH interacts with ALIX and thereby enhances ALIX mediated phenotypes in *Drosophila*.

**Results:**

In this study we identified ALIX as a POSH ubiquitination substrate in human cells: POSH induces the ubiquitination of ALIX that is modified on several lysine residues *in vivo *and *in vitro*. This ubiquitination does not destabilize ALIX, suggesting a regulatory function. As it is well established that ALIX rescues virus release of L-domain mutant HIV-1, HIV-1Δ_PTAP_, we demonstrated that wild type POSH, but not an ubiquitination inactive RING finger mutant (POSH^V14A^), substantially enhances ALIX-mediated release of infectious virions derived from HIV-1Δ_PTAP _L-domain mutant (YPX_n_L-dependent HIV-1). In further agreement with the idea of a cooperative function of POSH and ALIX, mutating the YPX_n_L-ALIX binding site in Gag completely abrogated augmentation of virus release by overexpression of POSH. However, the effect of the POSH-mediated ubiquitination appears to be auxiliary, but not necessary, as silencing of POSH by RNAi does not disturb ALIX-augmentation of virus release.

**Conclusion:**

Thus, the cumulative results identified ALIX as an ubiquitination substrate of POSH and indicate that POSH and ALIX cooperate to facilitate efficient virus release. However, while ALIX is obligatory for the release of YPX_n_L-dependent HIV-1, POSH, albeit rate-limiting, may be functionally interchangeable.

## Background

The release of nascent retrovirus particles requires the deployment of the ESCRT (endosomal sorting complex required for transport) to the site of virus budding at the cell surface [1-4]. ESCRT consists of four sub-complexes (ESCRT0-III) that normally reside on the cytoplasmic leaflet of the membrane of late endosomes where they function in tandem to target endocytosed plasma membrane proteins into mutivesicular bodies (MVBs) *en route *to lysosomes [5-7]. Recruitment of ESCRT to the site of virus budding is facilitated by interaction of specific ESCRT proteins with short peptide sequences termed late (L-) domains of retroviruses Gag proteins such as the p6 and p9 of human immunodeficiency virus (HIV)-1 and equine infectious anemia virus (EIAV), respectively.

ALIX (ALG2 interacting protein X) is a multifunctional protein adaptor that plays a central role in the regulation of intracellular protein trafficking and apoptosis. As an ESCRT-associated regulator of protein trafficking, ALIX plays an essential role in retrovirus release, an activity that is dependent on the interaction between the central V-domain and the L-domain consensus sequence YPX_n_L in Gag [8,9]. The function of ALIX to enhance virus release requires both the amino proximal Bro-domain that binds the ESCRT-III component CHMP4B as well as the carboxyl terminal proline-rich region (PRR) that interacts with multiple ALIX effectors including the tumor susceptibility gene (Tsg) 101 [9,10]. Tsg101 is a component of the ESCRT-I and is essential for the release of retroviruses like HIV-1 that contain a PTAP L-domain motif [1,11]. Based on the fact that ALIX binds both CHMP and Tsg101 and the redundancy of ESCRT-II for HIV-1 release [12], it has been proposed that ALIX facilitates virus budding by recruiting ESCRT-III to ESCRT-I to which the nascent virus initially binds. In agreement with this hypothesis, overexpression of the ALIX V-domain potently inhibits HIV-1 release, a dominant negative function that is reversed by mutations that abolish ALIX-Gag binding [13]. In contrast, preventing ALIX-Gag interaction has only a mild effect on the release of HIV-1 [14], while the ablation of Tsg101-Gag interaction blocks virus release almost completely [4,15-17].

In addition to the binding of HIV-1 p6, ALIX was recently found to bind to nucleocapsid (NC) *via *its N-terminal Bro-domain in an RNA independent manner [18]. This interaction depends on the zinc finger motif in NC which mediates packaging of genomic RNA into budding virions. Intriguingly, zinc finger mutant viruses exhibited a phenotype similar to PTAP mutants, suggesting a functional link between NC and the L-domain in p6 [18].

The *trans*-Golgi network (TGN) RING finger protein POSH (Plenty of SH3) is a scaffold protein that acts as an E3 ligase and as an activator of the JNK pathway (Jun N-terminal kinase), a function that is independent of its ubiquitination activity. Previously it was demonstrated that the ubiquitination activity of POSH is required for the trafficking of HIV-1 Gag from the TGN to the plasma membrane [19]. Subsequent reports implicated the E3 ligase activity of POSH in the degradation of the early endosome sorting factor Hrs (hepatocyte growth factor-regulated tyrosine substrate) [20], in the regulation of the *Drosophila *immune response by mediating degradation of the JNK activator TAK-1 (TGF-beta-activated kinase-1) [21] and in the regulation of calcium homeostasis through spatial control of HERP (homocysteine-inducible endoplasmic reticulum protein) [22].

Recent findings identified ALIX along with ALG-2 as binding proteins of POSH in *Drosophila*. Thereby, POSH enhances ALIX mediated phenotypes in transgenic fruit flies [23]. Most intriguingly, these findings provided a clue for a possible molecular mechanism by which POSH regulates virus release in human cells. In the present study, we therefore investigated whether POSH cooperates with ALIX in terms of promoting virus release. We hereby report that ALIX is a POSH ubiquitination substrate. Further, we demonstrate that E3 ligase active POSH substantially enhances ALIX-mediated virus release. However, down-regulation of POSH fails to block ALIX virus promoting activity, indicating that POSH is not obligatory but rather an auxiliary co-factor for ALIX to facilitate virus release.

## Results

### POSH binds to ALIX in human cells

**Figure 1:**
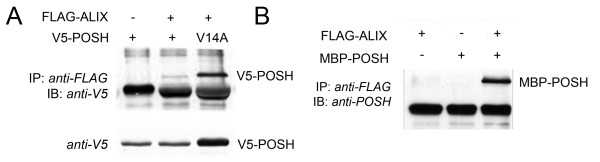
**POSH and ALIX are interacting proteins**. A. Interaction in mammalian cells. Flag-tagged ALIX and V5-tagged POSH-were co-expressed in 293-HEK cells. ALIX immune complexes were isolated from cell detergent extracts by immunoprecipitation with anti-FLAG and analyzed for the presence of POSH by Western blot with anti-V5. B. Direct interaction *in vitro*. Bacterially-expressed POSH (50 ng) and ALIX (50 ng) were incubated in binding buffer as described in Materials and Methods and then subjected to immonoprecipitation with anti-FLAG. Immune complexes were subsequently analyzed for the presence of POSH by Western blot with anti-POSH (PT1).

Previous results demonstrated an interaction between POSH and ALIX in *Drosophila*. To confirm this interaction in human cells, detergent extracts from HeLa cells transiently co-expressing epitope-tagged POSH and ALIX were subjected to immunoprecipitation of ALIX followed by Western blot analysis. POSH was efficiently co-precipitated by antibodies against ALIX (Figure 1A). Also the RING finger mutant POSH^V14A^, which is inactive as an E3 ligase and was previously shown to function in a dominant-negative manner [19], was efficiently co-immunoprecipitated using anti-ALIX antibodies (Figure 1A). The amount of ALIX-bound POSH was directly proportional to the amount of expressed POSH. Thus, POSH^V14A ^mutant that expresses at higher levels than wild-type POSH also shows increased binding to ALIX.

To ascertain that POSH binds directly to ALIX rather than through the mediation of another cellular factor, bacterially expressed recombinant POSH and ALIX were incubated *in vitro *and subsequently immunoprecipitated with antibodies against FLAG-tagged ALIX followed by Western blot analysis with POSH specific antibodies. Consistent with *in vivo *data, POSH was efficiently co-precipitated by antibodies against ALIX, indicating that POSH directly interacts with ALIX (Figure 1B).

### ALIX is a POSH ubiquitination substrate

**Figure 2:**
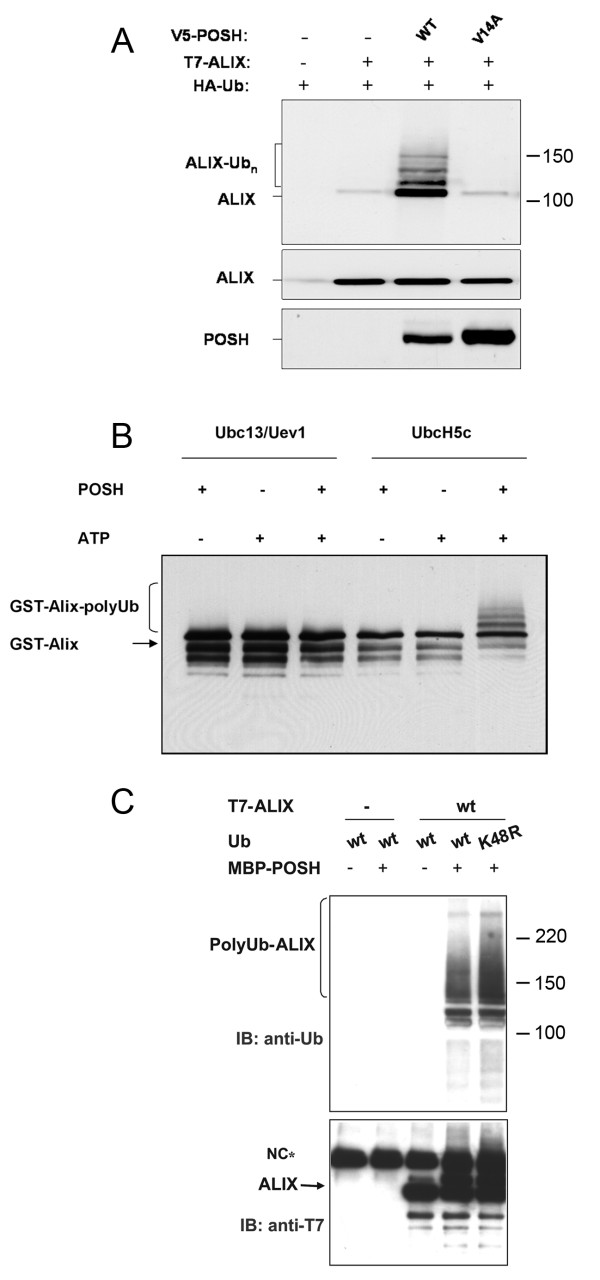
**ALIX is a POSH ubiquitination substrate**. A. Overexpression of POSH induces ALIX poly-ubiquitination. Flag-ALIX and V5-POSH were expressed in H310 cells together with HA-ubiquitin. ALIX immune complexes were isolated and ALIX-ubiquitination was detected by Western blot with anti-HA. B. POSH ubiquitinates ALIX *in vitro*. Bacterially expressed ALIX (50 ng) and POSH (40 ng) were incubated in an *in vitro *ubiquitination reaction with the indicated Ubc's (E2's) as described in Materials and Methods. ALIX was subsequently isolated by immunoprecipitation with anti-FLAG and analyzed by Western blot with anti-FLAG. C. ALIX-linked ubiquitination is independent of K48 residue in ubiquitin. T7-tagged-ALIX was overexpressed in HEK-293 cells. ALIX immune complexes were subsequently isolated by immunoprecipitation with anti-T7 and incubated in an *in vitro *ubiquitination reaction with POSH and UbcH5c and with either native ubiquitin or UbK48R as described in Materials and Methods. Following the ubiquitination reaction, ALIX was isolated by immunoprecipitation with anti-FLAG followed by a stringent wash with buffer containing 0.6% SDS (to disrupt ALIX-POSH interaction and thus ensure detection of ALIX-conjugated ubiquitin only).

The finding that ALIX associates with POSH prompted us to test whether ALIX is a POSH ubiquitination substrate. To investigate this we employed a HeLa H310A cell line in which the POSH gene is efficiently silenced due to constitutive expression of a POSH specific shRNA [22]. These cells were co-transfected with ALIX, HA-tagged ubiquitin and either RNAi insensitive *wt *POSH or POSH^V14A^. Subsequent analysis of immunoprecipitated ALIX by Western blot with anti-HA revealed that POSH, but not POSH^V14A^, induced the formation of high molecular weight ALIX-ubiquitin adducts (Figure 2A). It should be noted that the exogenous overexpression of POSH never exceeds the endogenous POSH levels and that of ALIX increases cellular protein level by approximately 10-fold (as determined by anti-ALIX, data not shown). Thus it is unlikely that the ubiquitination of ALIX by POSH is due to either non-physiological E3 levels or substrate concentration that could induce otherwise a non-physiological enzyme-substrate interaction.

To discern whether POSH ubiquitinates ALIX directly or rather, confers regulation of ALIX ubiquitination indirectly, we tested whether ALIX is a POSH ubiquitination substrate *in vitro*. To this end, bacterially expressed recombinant ALIX and POSH were incubated in the presence of ubiquitin, an E1 ubiquitin activating enzyme, an E2 conjugating enzyme and ATP in an *in vitro *ubiquitination reaction followed by Western blot analysis of the reaction products with anti-ALIX. The incubation of POSH with ALIX again produced high molecular ALIX species in an ATP-dependent fashion (Figure 2B). Together, the *in vivo *and *in vitro *ubiquitination results established that ALIX is indeed a POSH ubiquitination substrate.

The conjugation of multiple ubiquitin moieties to ALIX together with the fact that this ubiquitination does not induce ALIX degradation suggests a conjugation of either non-canonical poly-ubiquitin chains or multiple mono-ubiquitin molecules. The best characterized non-canonical protein polyubiquitination is that formed through ubiquitin moieties linked by isopeptide linkages via lysine (K) 63 residue of ubiquitin. This type of polyubiquitin chain formation is strictly dependent on the activity of the heterodimeric E2 Ubc13/Uev1 [24,25]. To gain insight into the type of ubiquitination of ALIX induced by POSH we sought to further characterize the ubiquitination by employing the *in vitro *ubiquitination assay. To this end, bacterially expressed recombinant ALIX and POSH were incubated in the presence of ubiquitin, an E1 ubiquitin activating enzyme, two different E2 conjugating enzymes (UbcH5c and Ubc13/Uev1) and ATP in an *in vitro *ubiquitination reaction followed by Western blot analysis of the reaction products with anti-ALIX. The incubation of POSH and ALIX with UbcH5c produced high molecular ALIX species in an ATP-dependent fashion (Figure 2B) while incubation with Ubc13/Uev1 did not although POSH is active with Ubc13 for ubiquitination of Herp [22]. As UbcH5c does not support formation of K63-linked polyubiquitin chains, the production of high molecular weight ALIX species is indicative of either multiple monoubiquitin molecules attached to multiple lysine residues within ALIX, or formation polyubiquitin chains linked by any other internal lysine of ubiqitin besides K63. Indeed, when a K48R ubiquitin, (where K48 is replaced by arginine), was employed in the *in vitro *reaction the polyubiquitinated ALIX species formed by POSH and UbcH5c were indistinguishable from those formed in the presence of native ubiquitin (Figure 2C). This result in conjunction with the inactivity of Ubc13/Uev1a indicates that POSH induces either poly-ubiquitin chains linked *via *any other lysine besides K48 and K63 in ubiquitin, or poly-mono-ubiquitination where single ubiquitin molecules are conjugated to multiple lysine residues of ALIX.

### Multiple ubiquitination sites are targeted by POSH

**Figure 3:**
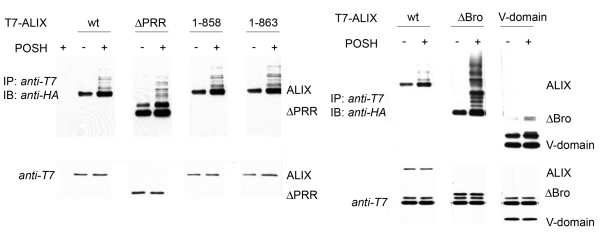
**POSH targets multiple sites in ALIX**. T7-tagged ALIX derivatives were expressed in HEK-293 cells and then subjected to immunoprecipitation with anti-T7. Isolated ALIX was subsequently incubated in an *in vitro *ubiquitination reaction and ALIX-ubiquitin conjugation was analyzed as described in Material and Methods and in the legend to figure 2.

We next set out to investigate which domain of ALIX is ubiquitinated by POSH. For that reason, truncation mutants of ALIX were co-expressed with POSH and HA-tagged ubiquitin in H310A cells and ALIX-ubiquitin adducts were determined by Western blot with anti-HA. The C-terminal PRR of ALIX was the most promising candidate to be targeted by POSH as it was shown to interact with SH3-domains [26]. However, deletion mutants of the last amino acids still were ubiquitinated by POSH (Figure 3). Even when the entire PRR was deleted, POSH still induced the formation of high molecular weight ALIX-ubiquitin adducts (Figure 3), indicating that POSH also binds and ubiquitinates regions outside the PRR. Also a deletion of the N-terminal Bro-domain did not disturb the POSH-ALIX interaction as an N-terminal truncation mutant of ALIX is still ubiquitinated by POSH (Figure 3). Only the central V-domain was not targeted by POSH, either for the reason that it does not contain an ubiquitination site, or that it does not bind to POSH (Figure 3).

### POSH enhances ALIX mediated augmentation of virus release

**Figure 4:**
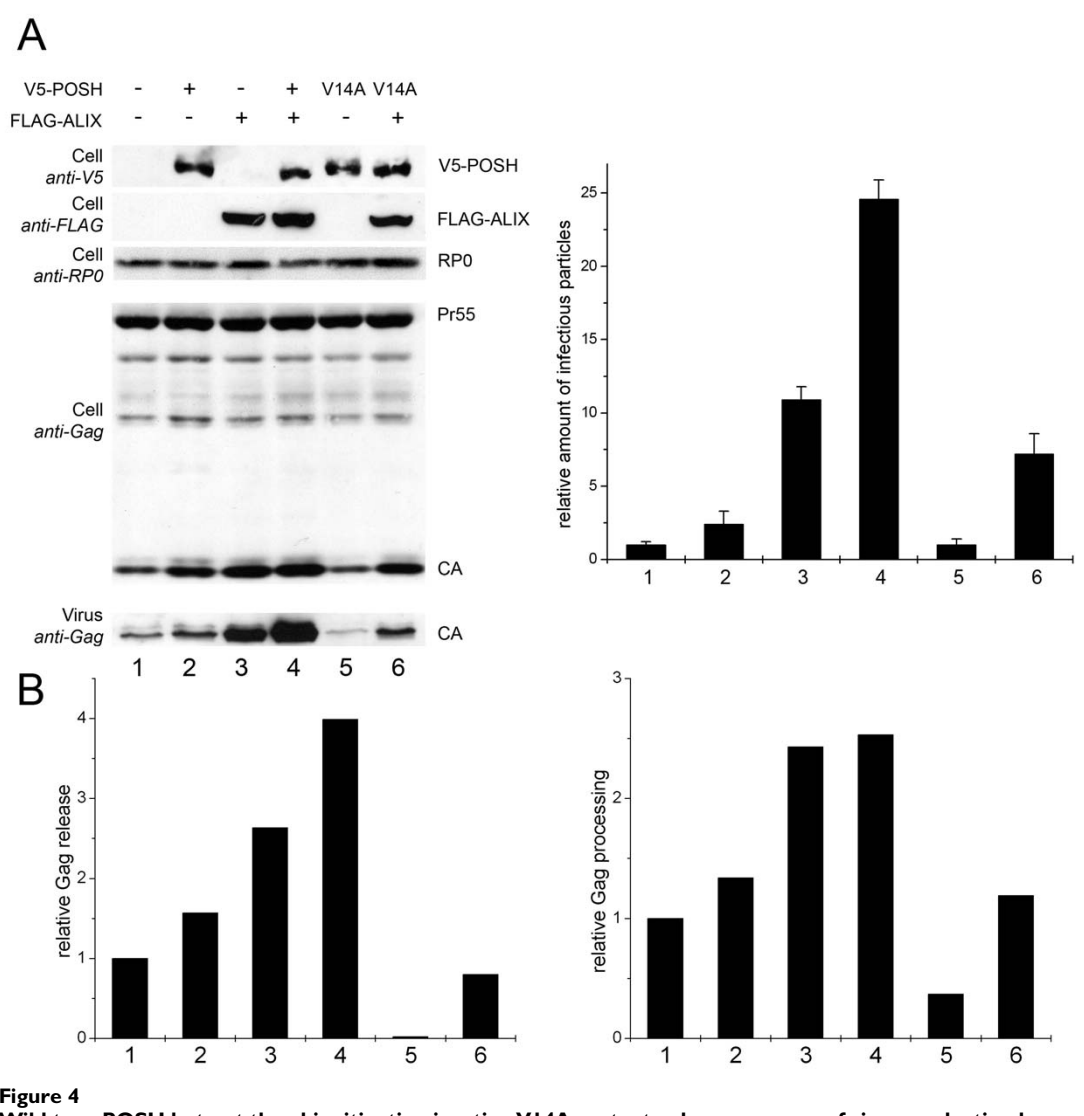
**Wild type POSH but not the ubiquitination inactive V14A mutant enhances rescue of virus production by ALIX**. A: The left panel shows Virus release, Gag processing and POSH and ALIXexpression analyzed by Western blot. The right panel shows the amounts of infectious units released from the cells analyzed by β-galactosidase quantification after infection of TZM-bl cells. Shown are virus infectivities relative to the HIV-1_ΔPTAP _control ± SD. HIV-1_ΔPTAP_-mutant was co-transfected with: lane 1, empty pcDNA3.1V5His; lane 2, a V5-POSH expression plasmid; lane 3, a FLAG-ALIX expression plasmid; lane 4, vectors expressing FLAG-ALIX and V5-POSH; lane 5, a plasmid encoding POSH^V14A^; lane 6, vectors encoding FLAG-ALIX and POSH^V14A^. RP0: ribosomal P0 antigen. B: Quantification of Western blot data using AIDA Software (Raytest). The left panel shows the amount of Gag release determined by the ratio of virus associated Gag/total Gag expressed relative to the control set to 1. The right panel shows the processing of Gag in the cell lysates as determined by the ratio of CA/total Gag expressed relative to the control set to 1.

Recent studies demonstrated that overexpression of ALIX can rescue the production of an HIV-1_ΔPTAP _mutant virus, that otherwise is severely attenuated due to its L-domain deficiency [9,14]. We therefore employed a full-length infectious HIV-1_NL4-3 _molecular clone, in which the PTAP motif was replaced by LIRL without affecting the *pol *ORF (HIV-1_ΔPTAP _[27]) to explore the function of ALIX in virus biogenesis. Consequently, HeLa SS6 cells were co-transfected with HIV-1_ΔPTAP _together with ALIX and POSH. Gag processing and release of infectious virions was determined 24-h post transfection by Western blot and single round infection of TZM-bl cells. The overexpression of POSH alone slightly enhanced both the expression of Gag and the release of virus particles (Figure 4, lane 2), while overexpression of ALIX dramatically stimulated virus release by approximately 10-fold (Figure 4, lane 3) as described previously [9]. However, co-expression of both exogenous POSH and ALIX further amplified virus production up to 25-fold compared to the control (Figure 4, lane 4), indicating that both POSH and ALIX regulate the release of L-domain mutant HIV-1 virions in a synergistic manner.

Measurement of virion associated CA and infectivity as indication of virus release seems to be over-simplistic in this case as it is evident that POSH expression also increased Gag processing (compare intracellular CA in lanes 1 and 2). Hence it is either that POSH contributed to virus release by increasing Gag expression (or stability) or that it functioned through a different mechanism. To discern between these possibilities we quantified the Western blot presented in figure 4A and subsequently determined the ratios between intracellular CA and virion CA and total Gag as measurements of Gag processing and virus release, respectively (Figure 4B). According to these measurements, expressed relative to the corresponding ratio in the control, POSH had a minor effect on Gag processing, while ALIX stimulated processing by approximately three-fold (Figure 4B, right panel). POSH in the presence of ALIX did not further stimulate Gag processing. Similarly, POSH minimally affected mutant virus release while ALIX stimulation was over 2.5-fold (Figure 4B, left panel). Yet, despite having no effect by itself, in the presence of ALIX, POSH further stimulated virus release up to 4-fold. Thus, these quantifications indicate that the synergistic contribution of POSH is through a mechanism independent of the increase of Gag expression.

As we found that POSH ubiquitinates ALIX, we consequently wanted to test whether the enhancing effect of POSH on virus release is dependent on its ubiquitination activity. To this end, the enzymatic inactive POSH^V14A ^mutant was tested for the ability to support virus release. Overexpression of POSH^V14A ^alone in HeLa SS6 cells did not influence overall virus production (Figure 4A, lane 5) but inhibited relative Gag processing and virus release indicating a dominant negative effect (Figure 4B, lane 5). In contrast to *wt *POSH, the POSH^V14A ^mutant did not further support ALIX mediated rescue of the HIV-1_ΔPTAP_. Moreover, consistent with a dominant negative effect, overexpression of POSH^V14A ^even slightly suppressed the stimulating effect of ALIX overexpression on virus production (Figure 4A and 4B, lane 6).

**Figure 5:**
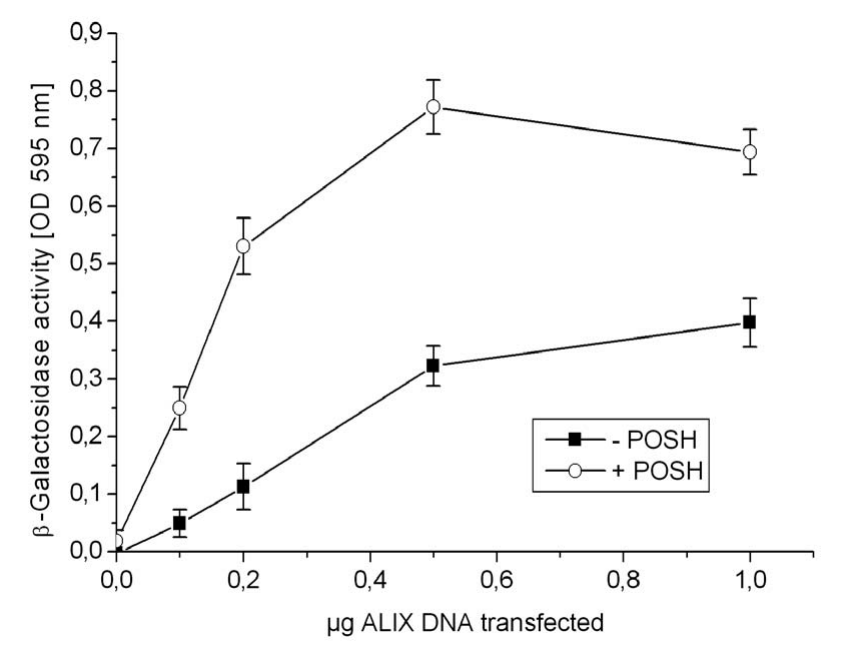
**POSH stimulates ALIX mediated virus release**. H310A cells were co-transfected with HIV_ΔPTAP_, increasing amount of ALIX plasmids, and either a control DNA (black squares) or a POSH expression vector (open circles). The amount of infectious units released from the cells was analyzed by β-galactosidase quantification after infection of TZM-bl cells.

Next we measured the requirement for POSH for HIV-1_ΔPTAP _release at varying ALIX concentrations. To this end H310A cells were co-transfected with HIV-1_ΔPTAP _and increasing amounts of ALIX expression plasmids co-expressed with either empty or POSH expression plasmid. Subsequent determination of virus release (Figure 5) showed that under conditions where ALIX was rate limiting, POSH consistently enhanced mutant virus release by approximately three fold. At higher concentration of ALIX, the enhancing effect of POSH was diminished, most likely due to saturation effects.

**Figure 6:**
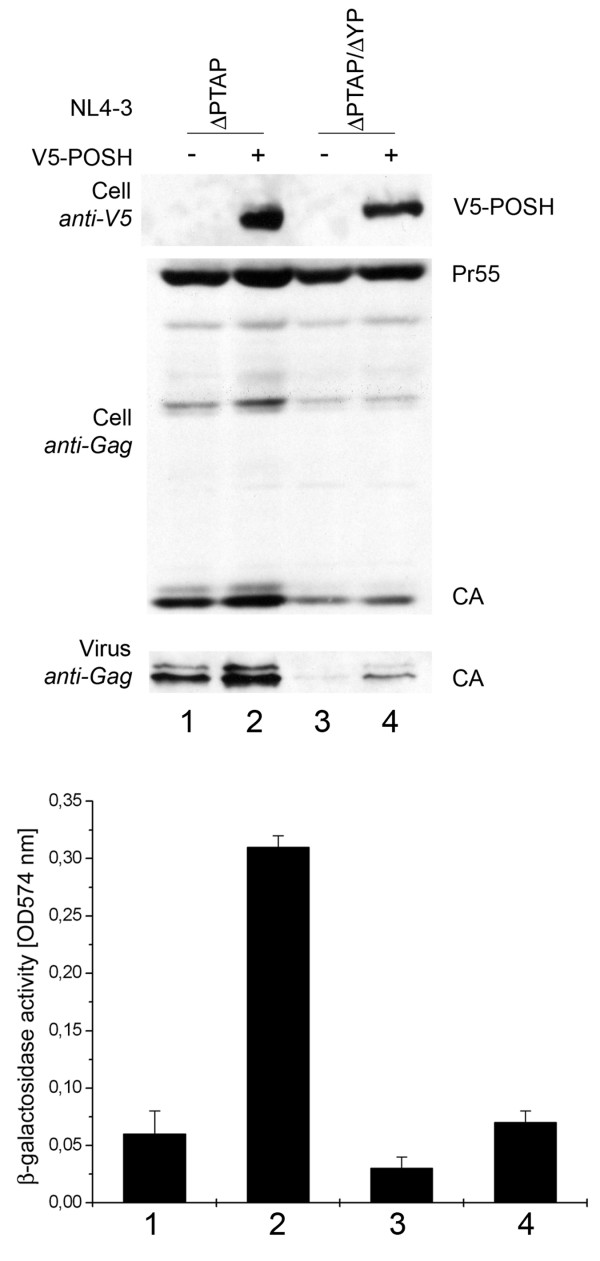
**POSH mediated enhancement of virus release requires a functional ALIX binding site in Gag**. Virus release, Gag processing, and exogenous expression of POSH were analyzed by Western blot (upper panel). Amount of infectious units released from the cells was analyzed by β-galactosidase quantification after infection of TZM-bl cells (lower panel). Shown are infectivities relative to the HIV-1_ΔPTAP _control ± SD. Lane 1 shows HIV-1_ΔPTAP _mutant co-transfected with the empty pcDNA3.1V5His control vector, lane 2 shows HIV-1Δ_PTAP _mutant co-transfected with a V5-POSH expression plasmid, lanes 3 and 4 show the HIV-1_ΔPTAP/ΔYP _double mutant co-transfected with the empty control plasmid and the vector expressing V5-POSH, respectively.

To further investigate the cooperative function of POSH and ALIX we tested whether the enhancing effect of POSH on virus release depends on the interaction between ALIX and HIV-1 Gag. Previous studies already indicated that POSH did not directly interact with HIV-1 Gag [19]. Consequently, it can be excluded that the stimulatory effect of POSH on virus release resulted from a direct interaction of POSH with Gag. Yet, abrogating the interaction between ALIX and Gag should provide further indication whether POSH requires ALIX to stimulate virus production. Therefore, we investigated the ability of POSH to enhance ALIX-dependent virus release of HIV-1_ΔPTAP _ALIX binding site (YPX_n_L) mutant, HIV-1_ΔPTAP/ΔYP_. To this end, POSH was co-expressed together with HIV-1 p6 ALIX binding site variants. The results depicted in figure 6 show that in the presence of the intact ALIX binding site in p6, POSH was able to enhance HIV-1_ΔPTAP _release (Figure 6, lanes 1 and 2). Consistent with previous results [9], the secondary ΔYP mutation further reduced HIV-1 release and infectivity by an additional 5-fold (Figure 6, lanes 1 and 3) as this mutation blocks ALIX-Gag interaction and as a result, the ability of ALIX to stimulate virus release. Overexpression of POSH had only slight effect on the release and infectivity of the HIV-1_ΔPTAP/ΔYP _double mutant virus, indicating that POSH requires the interaction of ALIX and Gag to substantially enhance virus production of a HIV-1_ΔPTAP _mutant.

### ALIX mediated rescue of HIV-1 L-domain mutant occurs independently of POSH

**Figure 7:**
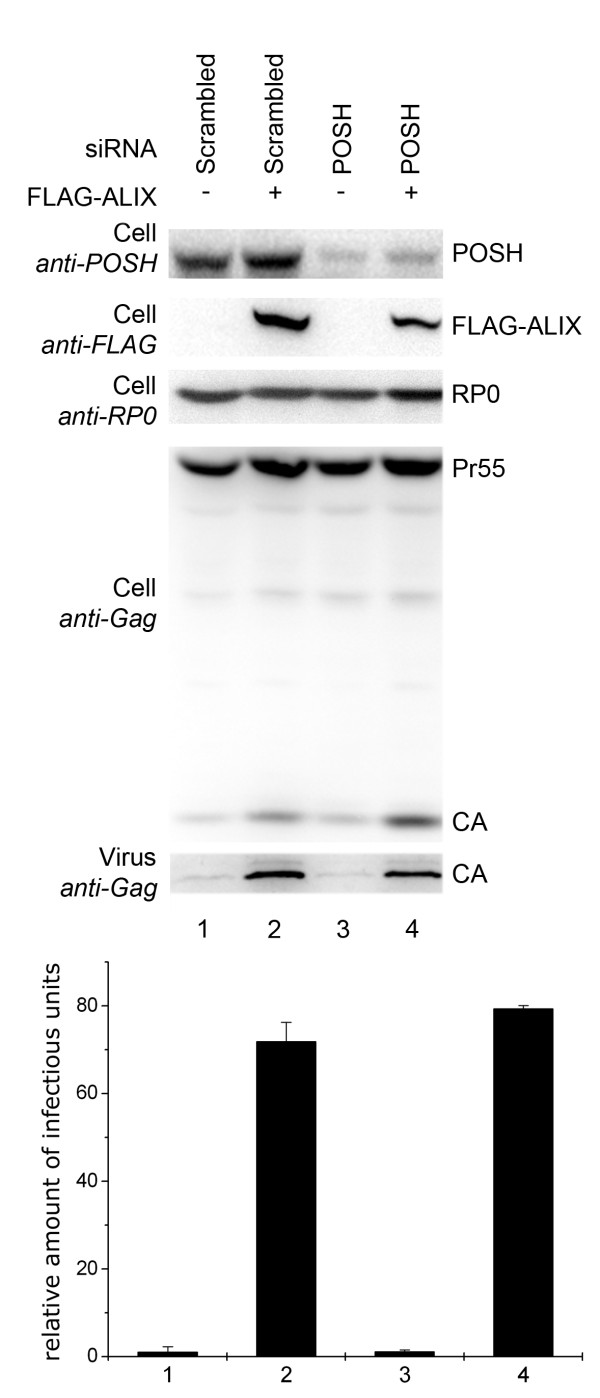
**ALIX mediated rescue of HIV-1 L-domain mutant occurs independently of POSH**. Endogenous levels of POSH were knocked down by RNAi in order to determine, whether ALIX can enhance virus release in the absence of POSH. HIV-1_ΔPTAP _was co-transfected with a control siRNA together with either an empty plasmid or an ALIX expression plasmid (lanes 1 and 2 respectively). HIV-1_ΔPTAP _was co-transfected with a POSH specific siRNA and either an empty plasmid or an ALIX expression plasmid (lanes 3 and 4 respectively). RP0: ribosomal P0 antigen.

Thus far, it could be demonstrated that POSH stimulated ALIX-mediated YPX_n_L-dependent virus release and that this effect is depended on its ubiquitination activity. To further elucidate the functional relationship between POSH and ALIX, we investigated the dependence of ALIX-mediated virus release on the ubiquitination by POSH. To address this question, endogenous levels of POSH were knocked down by RNAi in order to determine whether ALIX could enhance virus release in the absence of POSH. As shown in figure 7, significant (approximately 70%) knock down of endogenous levels of POSH by RNAi had no influence on ALIX-mediated rescue of the HIV-1_ΔPTAP _mutant, indicating that ALIX retained its activity even when endogenous POSH levels were reduced (Figure 7, compare lanes 1 and 2 to 3 and 4). Similar results were obtained when the POSH deficient H310A HeLa cell line was employed [22] and the ability of ALIX to rescue the release HIV-1 L-domain mutants in these cells was compared to the control cell line H314A (data no shown). Hence, it appears that the virus release enhancing function of ALIX can be stimulated by POSH-mediated ubiquitination, while in contrast down-regulation of POSH does not significantly influence the ALIX activity.

## Discussion

ALIX is a proline rich, multifunctional protein that has been implicated in numerous cellular functions such as regulation of apoptosis and endocytotic protein trafficking [26,28]. ALIX was shown to associate with the MVB pathway and thereby being involved in budding of enveloped viruses. More recently it was demonstrated that ALIX as an ESCRT-III associated protein is also involved in the terminal stages of cytokinesis, where it is recruited to the midbody through binding to Cep55 (centrosome associated protein 55) [29,30]. Biogenesis of intra-endosomal vesicles in MVBs, midbody abscission during cytokinesis and virus budding are topologically equivalent inasmuch as the final step, the pinching off the membrane-stalk, is regulated by the ESCRT.

Due to its multitasking nature, the function of ALIX is tightly regulated by its interaction partners. As already demonstrated earlier by others, ALIX is phosphorylated by the Src thyrosine kinase, which regulates its association with membranes and cytoskeleton as well as its affinity to binding partners SETA/CIN85 and Pyk2 that are involved in receptor downregulation and cell adhesion, respectively [31].

In this study we propose a role for ubiquitination in the regulation of ALIX function. We found, that the TGN-associated scaffolding protein POSH, which exhibits E3-ligase activity mediated by its N-terminal RING-domain, ubiquitinates ALIX. Intriguingly, this ubiquitination does not induce proteasomal degradation of ALIX, as there was no reduction in the level of intracellular ALIX upon POSH overexpression. Moreover, as it could be excluded that the ubiquitin chains are linked *via *K48 of ubiquitin, the non-canonical ubiquitination apparently has a regulatory function. While it could be established that POSH targets multiple lysine residues in ALIX, the detected high molecular ALIX species possibly arise from conjugation of monomeric ubiquitin moieties to several lysine residues. ALIX contains altogether 65 lysine residues from which 33 were predicted by computational analysis to be accessible for ubiquitination (ubiquitin nuggets, ). However, the exact nature of this ubiquitination remains obscure. Given that ALIX is ubiquitinated in the presence of UbcH5c but not in the presence of Ubc13/Uev1 it is likely that POSH either induces multi-ubiquitination on several lysine residues within ALIX or poly-ubiquitin chains linked via ubiquitin lysine residues other than K48 and K63 (*e. g. *K6, K11, K27, K29, and K33) as has been recently described [32]. The functional significance of these types of ubiquitinations on target proteins is still poorly understood.

Besides its numerous functions in regulating cellular pathways, ALIX was described as binding partner of HIV-1 p6 and EIAV p9 thereby being involved in budding and release of these retroviruses [8,9]. Previously it was demonstrated that the ubiquitination activity of POSH is required for HIV-1 particle production by facilitating Gag trafficking from the TGN to the cell membrane and thus, stimulates the release HIV-1 particles [19]. The finding that there is a functional interaction between POSH and ALIX prompted us to investigate the importance of this interaction for virus release. Though the specific regulation conferred on ALIX through POSH-dependent ubiquitination is unclear, the data indicates that both proteins somehow cooperate to promote virus release: Overexpression of POSH substantially stimulated the ALIX mediated rescue of an HIV-1_ΔPTAP _mutant, the stimulatory effect of POSH on virus production was blocked by abrogating Gag-ALIX interaction through introduction of specific mutations within Gag that prevented ALIX binding and the enhancing effect of POSH was completely abolished when its ubiquitination activity was disrupted due to a RING finger mutation (POSH^V14A^). However, the minor residual activity of POSH observed when ALIX-Gag interaction was blocked by the ΔYP mutation in the L-domain containing p6 Gag protein might indicate that POSH also regulates another factor along the pathway that mediates virus release. This together with the fact that siRNA-mediated knockdown of POSH fails to block the function of ALIX raises the question of whether the cooperative function of POSH and ALIX in terms of promoting virus release is a result of a direct interaction between both proteins, or a consequence of an independent positive role of POSH-mediated ubiquitination somewhere else in the pathway. The observation that POSH, which by itself does not interact detectably with Gag, ubiquitinates ALIX is consistent with a model whereby the binding of Gag to ALIX meditates virus egress while POSH acts as an auxiliary factor. Since downregulation of POSH has no detectable influence on the function of ALIX, it is either that POSH is not strictly required or that the POSH function can be substituted by another E3-ligase. Similar to Tsg101, which is also substrate for two different E3 ligases, namely Tal (Tsg101 associated ligase) and Mahogunin [33,34], ALIX may be ubiquitinated by an additional E3 ligase, when POSH is downregulated.

Until now, the molecular mechanism by which ALIX supports virus release is unresolved. Apparently, it requires the interaction of the N-terminal Bro-domain of ALIX with the ESCRT-III component CHMP4B as mutations in ALIX that prevent ALIX/ESCRT-III interaction abolish the ability of ALIX to rescue HIV-1 L-domain mutants [9,10]. In addition, the C-terminal PRR of ALIX was shown to be essential for virus release [9,10], however, the nature of this activity is currently unknown. Furthermore, as ALIX fulfills several distinct cellular functions, it is possible that ubiquitination by POSH regulates a function that is redundant for virus release. That the ubiquitination of ALIX by POSH might not be essential for virus release is supported by the results that show lack of correlation between the requirement for ALIX ubiquitination and for the capacity of ALIX to support virus release. For example, ALIX truncation mutants that cannot promote virus release such as the ΔBro and the ΔPRR are similarly ubiquitinated and both ubiquitination patterns are similar to that of native ALIX.

Previous studies implicated POSH as a regulator of Gag transport from the TGN to the cell membrane, a function that precedes ESCRT-mediated virus budding from the membrane. The function of POSH mediated ubiquitination of ALIX may accelerate a step of Gag transport prior to the virus assembly at the cell membrane. As the rate-limiting step of virus production of HIV-1_ΔPTAP _mutant virions is the ESCRT-dependent release of virus particles from the cell membrane, inhibition of steps earlier in the pathway would not show a significant phenotype as long as enough Gag is transported to the cell membrane.

However, besides its role in ESCRT, ALIX was initially identified as interaction partner of ALG-2, a calcium binding protein that is necessary for induction of apoptosis [35,36]. The observation that POSH binds both, ALIX and ALG-2 in a calcium dependent manner [23] together with the implicated role of POSH in the induction of apoptosis and neuronal differentiation [37-39] suggest a functional role for POSH and ALIX in the regulation of apoptosis and calcium metabolism. Further evidence for this hypothesis comes from findings that POSH regulates calcium homeostasis through spatial control of HERP [22]. The fact, that ALIX links ALG-2 and ESCRT pathways [40] might indicate that these pathways are somehow connected and are utilized by HIV-1.

It has been reported that actin polymerisation is essential for Gag transport of EIAV to the plasma membrane and the subsequent disassembly of actin filaments facilitates virus budding and virion release from the cell membrane [41]. ALIX, that contains actin and alpha actinin binding sites, also plays a pivotal role in actin organisation. Pan and colleagues reported that ALIX expression in a fibroblast cell line was required to maintain actin-based structures such as stress fibers and lamellipodia [42].

We have no evidence for direct interaction between POSH and actin. Nevertheless, considering the interaction between POSH and ALIX, the actin interaction properties of ALIX and the requirement for actin dynamics for virus production and release, we propose (a model) whereby both proteins cooperate at the plasma membrane where ALIX serves a dual function: it bridges between Gag and actin through respective binding sites while it recruits ESCRT to initiate virus budding. POSH may regulate disassembly of actin from Gag, possibly through ubiquitination of ALIX, thereby facilitating detachment of nascent virus particles from the cell membrane.

The model is consistent with the absolute requirement for ALIX as it is essential for mutant virus budding. Likewise, the model is in agreement with overexpression of POSH enhancing virus release possibly by increasing the rate of actin disassembly.

Finally, it should to be considered that POSH also interacts with another ESCRT-associated protein as it was shown to ubiquitinate Hrs, which in contrast to ALIX, is subsequently targeted for proteasomal degradation. Hrs interacts with the ESCRT-I components Tsg101 and HCRP1/Vps37A and C-terminal fragments of Hrs that interact with Tsg101 interfere with HIV-1 particle production [43]. Thus, it has to be considered that overexpression as well as downregulation of POSH may also regulate HIV-1 release distant to the hereby described POSH-ALIX interaction and overlapping effects may account for the partial nature of the phenotype described.

## Conclusion

We have identified ALIX as an ubiquitination substrate of POSH and indicate that both proteins are involved in the process of YPX_n_L-dependent HIV-1 release. However, while ALIX is obligatory for the release of HIV-1 L-domain mutant viruses, POSH, albeit rate-limiting, may be functionally interchangeable for the function of ALIX.

## Methods

### Plasmids and siRNA

The HIV-1_ΔPTAP _mutant is based on the HIV-1 molecular clone NL4-3 [44] in which the ^7^PTAP^10 ^L-domain motif was replaced by ^7^LIRL^10 ^without affecting the overlapping *pol*-reading frame [27]. In the HIV-1_ΔPTAP/ΔYP_, in addition to the PTAP domain, the ^36^YP^37 ^of the ^36^YPX_n = 3_L^41 ^ALIX binding motif in p6 was mutated to ^36^SR^37 ^by site directed mutagenesis, without affecting the overlapping *pol*-reading frame. Plasmids pcDNA3.1-POSH-V5-His and pcDNA3.1-POSH-V14A-V5-His were described previously [19]. Plasmids containing ALIX cDNA were derived from IMAGE clone IMAGE: 4340998. For expression in mammalian cells, the 5' region of the cDNA was PCR amplified in frame with either T7 tag or a FLAG tag peptide sequence, and ligated back into the same plasmid to create pCMV-T7-ALIX and pCMV-FLAG-ALIX. For bacterial expression FLAG-ALIX or a region containing residues 200–320 were cloned into pGEX-6P (GE Healthcare). To the full-length ALIX an N-terminal six histidine tag was also added. The short interfering RNA (siRNA) against POSH was described in [19]. The H310A cell line that stably express POSH-specific short hairpin (sh)RNA was previously described [22].

### Production of recombinant ALIX

For expression of recombinant full-length ALIX, GST-FLAG-ALIX-6xHIS was induced with IPTG at 22°C for 3 h. Protein was purified by glutathione chromatography dialyzed into 20 mM Tris-HCl pH 7.6, 100 mM NaCl, 20 mM Imidazole, 10 mM β-mercaptoethanol and further purified by Ni-NTA (Qiagen) chromatography and further dialyzed into 20 mM Tris pH 7.6, 1 mM DTT, 0.5 mg/ml ovalbumin.

### Antibodies

Antibodies against FLAG and V5 were obtained from Sigma and Invitrogen, respectively. Ubiquitin monoclonal antibody FK2 was from mouse anti-POSH monoclonal antibody was previously described [22]. The human anti-HIV-1 Gag antiserum was provided by the AIDS Research and Reference Reagent Program. For anti-ALIX sera preparation GST-ALIX (200–320) was expressed in E. coli BL21 by IPTG induction. Protein was purified from cell lysate by glutathione chromatography and the GST moiety removed by digestion with PreScission protease (GE Healthcare). The isolated polypeptide was used to raise antibodies in rabbits (Sigma-Aldrich, Israel).

### Cell culture

HEK-293T, HeLa SS6, H310A, H314A [22], and TZM-bl cells were cultured in Dulbecco's modified Eagle's medium (DMEM) supplemented with 10% inactivated fetal calf serum (FCS), 2 mM L-glutamine, 100 U/ml penicillin and 100 μg/ml streptomycin. H314A and H310A cells were cultured in the same medium but additionally supplemented with 0.5 mg/ml hygromycin Jurkat T-cells were maintained in RPMI 1640 supplemented with 10% inactivated FCS, 2 mM L-glutamine, and 100 U/ml penicillin and 100 μg/ml streptomycin.

### *In vitro *ALIX ubiquitin conjugation assays

Recombinant ALIX: Purified recombinant ALIX (50 ng) was incubated with 50 nM of recombinant E1, 0.3 μM of UbcH5c or Ubc13/Uev1a (where indicated), 13 nM of bacterially expressed maltose-binding protein (MBP)-POSH [19] in a final volume of 25 μl containing 40 mM Tris-HCl pH 7.5, 1 mM DTT, 2 mM ATP, 5 mM MgCl_2_, 5 × 10^-3 ^(vol/vol) Tween 20 and 2 μg ubiquitin. After incubation for 45 min at 30°C, ALIX was isolated via immunoprecipitation. The ALIX immune complexes were washed in a buffer containing 0.6% SDS (to dissociate POSH) and ubiquitin conjugation was subsequently detected by Western blot with anti-ubiquitin (FK2).

Cell-expressed ALIX: 3 μg of T7-tagged ALIX or ALIX truncation mutants expressing plasmids (as indicated in the figure legends) were transfected each into HEK-293 cells (5.4 × 10^6 ^cells per 10 cm dish). One day after the transfection, ALIX was immunoprecipitated with anti-T7 and protein G beads. One third of the ALIX beads packed volume was transferred into a fresh tube and subsequently incubated in an *in vitro *ubiquitination reaction as described above. ALIX ubiquitination was detected by western blot analysis with anti-ubiquitin as described above.

### *In vivo *ALIX ubiquitination

HeLa SS6 cells or H310 cells (9 × 10^5 ^cells in a 6-well plate) were transfected with T7-ALIX plasmid (0.5 μg) and V5-POSH (1 μg) or V5-POSH V14A (0.2 μg) and HA-tagged ubiquitin (1 μg). The day after the transfection ALIX was isolated by immunoprecipitation with anti-T7. Ubiquitination of ALIX was detected by Western blot analysis of the ALIX immune complexes with anti-HA.

### ALIX-POSH binding assays

Interaction in cells: FLAG-tagged ALIX (3 μg) and V5-POSH (6 μg) or V5-POSH V14A (1.2 μg) were co-expressed in HEK 293 cells. One day after the transfection cells were extracted in lysis buffer containing 10 mM Tris HCl pH 7.5, 150 mM NaCl, 5 mM MgCl_2 _and 1% Octyl β-D-glucopyranoside (Ultrol grade, Calbiochem). ALIX was isolated by immunoprecipitation with anti-FLAG beads. The beads were washed three times with lysis buffer and subsequently resolved in a 7.5% SDS gel. ALIX-bound POSH was detected by Western blot analysis with anti-V5.

Interaction between bacterially-expressed ALIX and POSH: Bacterially expressed GST-Flag-ALIX and POSH (50 ng each) were incubated in lysis buffer as described above. After incubation for at 30°C for 45 min. ALIX was isolated by immunoprecipitation as described above. The ALIX beads were washed three times with lysis buffer and then subjected to Western blot analysis with anti-POSH (PT1).

### Virus release and infectivity assays

HeLa SS6, H310A and H314A cells (9 × 10^5 ^cells/well in 6-well plates) were transfected with 1 μg of HIV-1_ΔPTAP _plasmid together with 0.2 μg of ALIX expression vector, 1 μg POSH expression vector and 0.2 μg POSH^V14A ^expression vector per well. DNA content was adjusted to 4 μg total DNA by addition of empty pcDNA3.1V5His and transfection was carried out with 10 μl Lipofectamine™ 2000 (Invitrogen) per well according to manufacturer's instructions. For RNAi HeLa SS6 (2 × 10^5 ^cells/well) cells were initially transfected with siRNA using Lipofectamine™ 2000 according to manufacturer instructions and split the next day. 48 h after the initial transfection, cells were co-transfected with another portion (one fourth of first transfection) of siRNA together with plasmids encoding ALIX (0.1 μg), POSH (1 μg), V14A (0.2 μg) and HIV-1_ΔPTAP _(1 μg). Cells and virions were harvested 24 h post transfection. Proteins were extracted by RIPA (1% NP-40, 0.5% Na-DOC, 0.1% SDS, 0.15 M NaCl; 50 mM Tris-HCl pH 7.4; 5 mM EDTA) lysis and analyzed by Western blotting. Cell debris from virus containing supernatant was removed by centrifuging at 1000 × *g *for 5 min and 8000 × *g *for 10 min. Virions were subsequently pelleted over 20% sucrose at 20000 × *g *for 90 min, re-suspended in 1 ml of PBS, pelleted again for 20000 × *g *for 90 min to remove serum albumins and finally analyzed by Western blotting. HIV-1-infectious titers were assayed by single round infection of Hela TZM-bl cells. 4000 cells per well in 96 well plate were infected by overnight incubation with prepared virus supernatants in a total volume of 100 μl in the presence of 10 μg/ml polybrene. To avoid spread of infection, cells were washed and dextran-sulfate (100 μg/ml) was added 16 h post infection. Three days post infection, cells were lysed in 40 μl CAT lysis buffer (Roche) and β-galactosidase activity was determined. Therefore, cell 35 μl of the cell lysate were added to 215 μl Z buffer pH 7.0 (60 mM Na_2_HPO_4_; 40 mM NaH_2_PO_4_; 10 mM KCl; 10 mM MgSO_4_; 0.05 mM β-mercaptoethanol; 0,08% SDS) containing 0.375 mg chlorophenolred β-D-galactopyranoside (Roche), incubated at room temperature 1–8 h and read at 574 nm.

## Authors' contributions

YR initiated the project and US initiated and supervised the infectious experiments. OF and EY carried out binding and ubiquitination experiments. DT supervised the molecular biology-associated work. OE contributed important scientific insight. JV analyzed virus release experiments assisted by TW and SS in many procedures. NB did the electron microscopy (data not shown). JV and YR wrote the manuscript.
